# Cardiac Injury in COVID-19: A Systematic Review of Relevant Meta-Analyses

**DOI:** 10.31083/j.rcm2312404

**Published:** 2022-12-12

**Authors:** Konstantinos G Kyriakoulis, Ioannis G Kyriakoulis, Ioannis P Trontzas, Nikolaos Syrigos, Ioanna A Kyprianou, Eleni Fyta, Anastasios Kollias

**Affiliations:** ^1^National and Kapodistrian University of Athens, School of Medicine, Third Department of Medicine, Sotiria Hospital, 11527 Athens, Greece

**Keywords:** COVID-19, cardiac injury, prognosis, mortality, meta-analysis

## Abstract

**Background:**

Cardiac injury (CI) is not a rare condition among hospitalized patients with coronavirus disease 2019 (COVID-19). Its prognostic value has been extensively reported through the literature, mainly in the context of observational studies. An impressive number of relevant meta-analyses has been conducted. These meta-analyses present similar and consistent results; yet interesting methodological issues emerge.

**Methods:**

A systematic literature search was conducted aiming to identify all relevant meta-analyses on (i) the incidence, and (ii) the prognostic value of CI among hospitalized patients with COVID-19.

**Results:**

Among 118 articles initially retrieved, 73 fulfilled the inclusion criteria and were included in the systematic review. Various criteria were used for CI definition mainly based on elevated cardiac biomarkers levels. The most frequently used biomarker was troponin. 30 meta-analyses reported the pooled incidence of CI in hospitalized patients with COVID-19 that varies from 5% to 37%. 32 meta-analyses reported on the association of CI with COVID-19 infection severity, with only 6 of them failing to show a statistically significant association. Finally, 46 meta-analyses investigated the association of CI with mortality and showed that patients with COVID-19 with CI had increased risk for worse prognosis. Four meta-analyses reported pooled adjusted hazard ratios for death in patients with COVID-19 and CI vs those without CI ranging from 1.5 to 3.

**Conclusions:**

The impact of CI on the prognosis of hospitalized patients with COVID-19 has gained great interest during the pandemic. Methodological issues such as the inclusion of not peer-reviewed studies, the inclusion of potentially overlapping populations or the inclusion of studies with unadjusted analyses for confounders should be taken into consideration. Despite these limitations, the adverse prognosis of patients with COVID-19 and CI has been consistently demonstrated.

## 1. Introduction

Cardiac injury (CI) is not a rare phenomenon among hospitalized patients with coronavirus disease 2019 (COVID-19) [1, 2, 3]. Its definition involves the increase of cardiac biomarkers levels, and it has been most commonly defined as an increase in cardiac troponin levels above the 99th percentile upper reference limit [1, 2, 3]. CI is more frequent among severe and critically ill patients [2, 3, 4] and serves as a prognostic factor for poor COVID-19 related outcomes and increased mortality [2, 3, 5, 6].

The exact mechanisms of CI in patients with COVID-19 are not well understood and clearly established. High troponin levels may be attributed to a variety of conditions affecting cardiac function (e.g., type 2 myocardial infarction, myocarditis, stress cardiomyopathy, arrhythmia, pulmonary embolism) and do not necessarily indicate a true type 1 myocardial infarction [2, 7]. Based on these considerations, the American College of Cardiology commented early on during the pandemic on the use of cardiac biomarkers in patients with COVID-19 and advised “*to only measure troponin or natriuretic peptides if the diagnosis of acute myocardial infarction or heart failure are being considered on clinical grounds*” [8]. The rational of this recommendation was that in many cases resources will be wasted and risk of exposure will be unacceptably high, seeking a type 1 myocardial infarction that is far less common (prevalence among patients with COVID-19 not still defined) than the multifactorial non-atherosclerotic CI (prevalence in hospitalized patients with COVID-19 about 21% [1]).

Cardiac involvement has been a major concern in COVID-19, and subsequently this recommendation has been recently slightly modified, with investigation for cardiac involvement (including troponin levels measurement) recommended in case of “*symptoms suggestive of cardiac involvement, including chest pain/pressure, dyspnea, palpitations, and syncope*” [3]. Interestingly, according to the recent consensus document by the European Society of Cardiology (ESC): “*as in patients without COVID-19, cardiac troponin T/I concentrations should be measured whenever, on clinical grounds, type 1 myocardial infarction is suspected*” [2]. According to the ESC, troponin levels may offer some prognostic information, however better prognostic tools have been developed and the risk of inappropriate diagnostic or therapeutic interventions may increase [2].

Since the COVID-19 outbreak, an impressive number of meta-analyses have been conducted aiming to investigate the incidence of CI and its impact on clinical outcomes of COVID-19 hospitalized patients. The aim of the current systematic review is to identify and summarize these relevant meta-analyses.

## 2. Materials and Methods

### 2.1 Search Strategy

A systematic PubMed search was conducted in line with PRISMA recommendations independently by two investigators (KGK and IPT) [9]. Literature search was conducted using the algorithm *(“coronavirus 2019” OR “2019-nCoV” OR “SARS-CoV-2” OR “COVID-19” OR “coronavirus disease 2019”) AND (troponin OR “cardiac injury” OR “myocardial injury”) AND (“meta-analysis” OR metaanalysis)* until May 04, 2022. Articles were also selected from references of relevant articles and by hand search. Disagreements were resolved by consensus with a senior author (AK).

### 2.2 Selection of Studies

Eligible studies were full-text meta-analysis articles in English that investigated: (i) the incidence of CI among COVID-19 hospitalized patients, and/or (ii) the association and impact of CI on COVID-19 infection severity and/or mortality. Studies on the impact of CI on COVID-19-related outcomes used two different approaches/kind of analyses: (i) comparison/prediction of outcome in CI vs non-CI patients [odds ratio (OR), relative risk (RR) and hazard ratio (HR) used as outcomes of interest in this case], and (ii) comparison/difference of cardiac biomarker levels (e.g., troponin) in mild vs severe disease, severe vs critical disease or survivors vs non-survivors depending on the population included in each study. Outcomes of interest in the latter case were measures of pooled difference between the two comparison groups [e.g., standardized mean difference (SMD), weighted mean difference (WMD)].

Meta-analyses on pediatric populations were excluded. In meta-analyses that included studies based on different definitions of CI, data based on troponin level were deemed more suitable for extraction. In studies where results were reported based on both troponin level (as a continuous variable) and CI status (as a dichotomous variable based on troponin cutoffs), data on CI as a dichotomous variable were extracted.

### 2.3 Data Extraction

Data concerning the aims and outcomes of interest, the literature search period, the number of included studies and patients, the CI definition and the main findings of each included meta-analysis were extracted, tabulated and reviewed by all authors.

## 3. Results

**Fig. 1.**
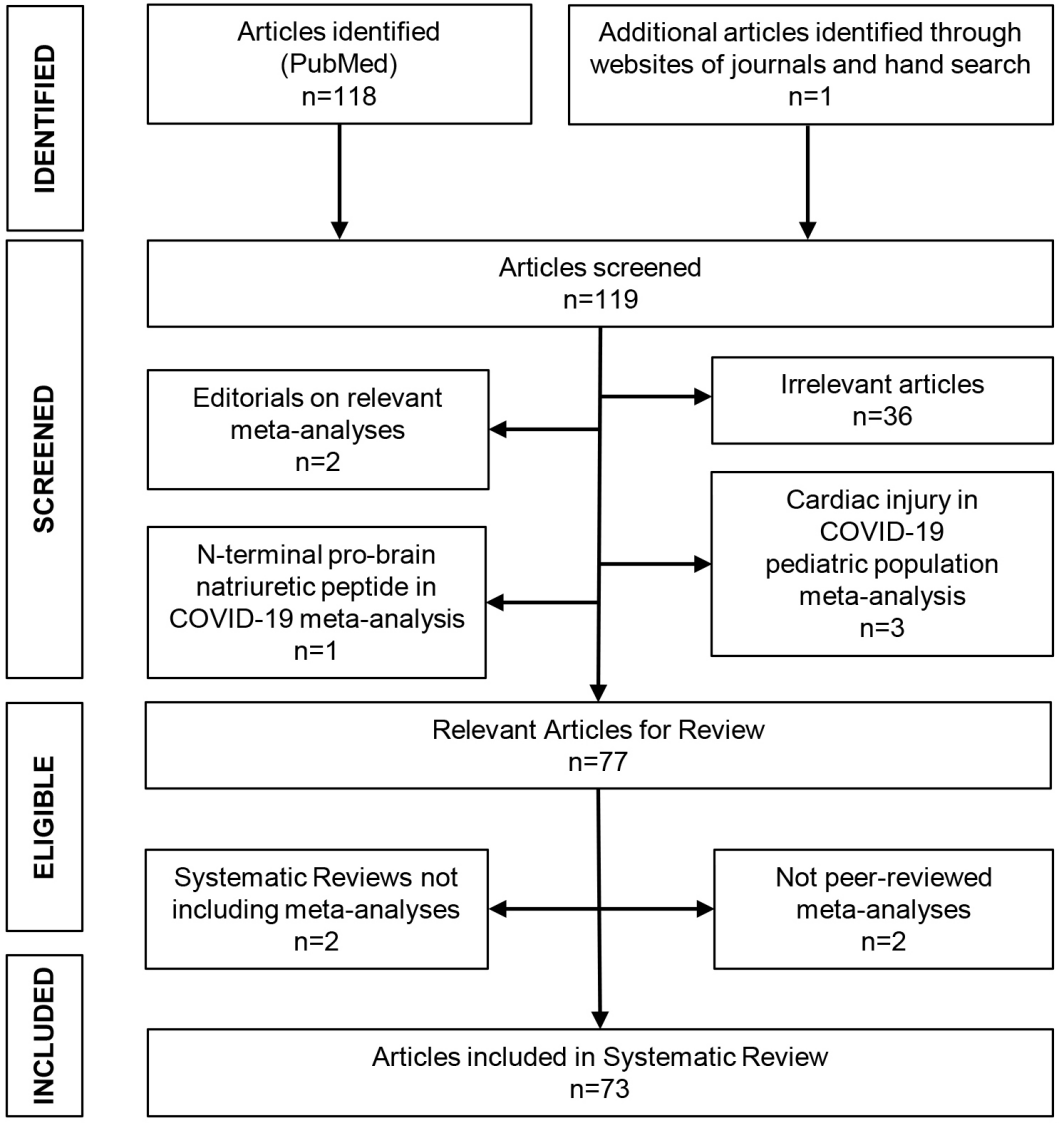
**Flowchart for the selection of the included studies**.

**Fig. 2.**
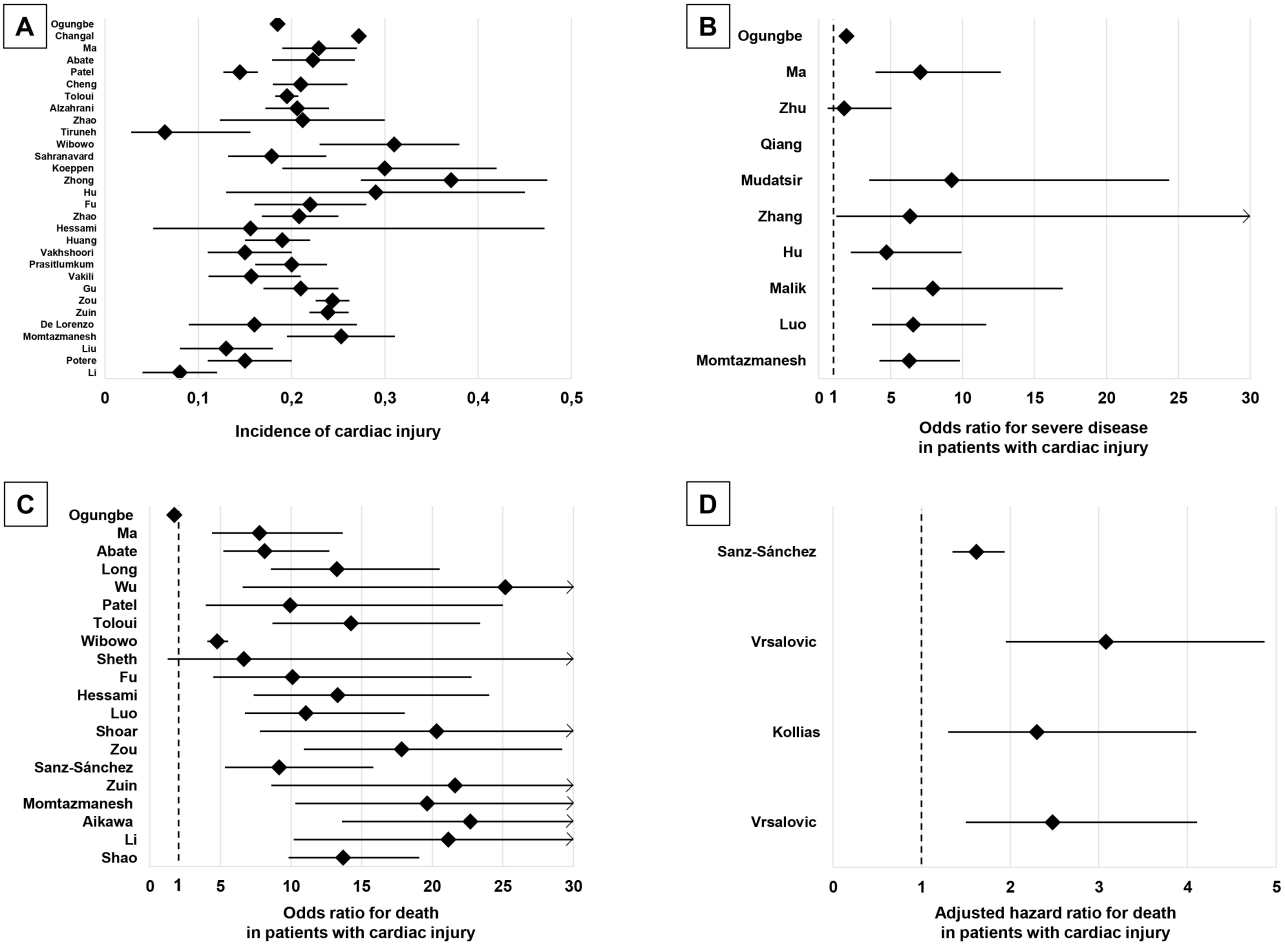
**Graphical summary of the main findings of the present systematic review**. (A) Meta-analyses reporting incidence of cardiac injury. (B) Meta-analyses reporting odds ratio for severe disease in patients with cardiac injury. (C) Meta-analyses reporting odds ratio for mortality in patients with cardiac injury. (D) Meta-analyses reporting adjusted hazard ratio for mortality in patients with cardiac injury.

Among 118 articles initially retrieved, 73 fulfilled the inclusion criteria and were included in the systematic review [1, 4, 5, 10, 11, 12, 13, 14, 15, 16, 17, 18, 19, 20, 21, 22, 23, 24, 25, 26, 27, 28, 29, 30, 31, 32, 33, 34, 35, 36, 37, 38, 39, 40, 41, 42, 43, 44, 45, 46, 47, 48, 49, 50, 51, 52, 53, 54, 55, 56, 57, 58, 59, 60, 61, 62, 63, 64, 65, 66, 67, 68, 69, 70, 71, 72, 73, 74, 75, 76, 77, 78, 79]. The search strategy and flowchart for the selection of studies are shown in Fig. 1. Main characteristics of the included studies are presented in Table 1 (Ref. [1, 4, 5, 10, 11, 12, 13, 14, 15, 16, 17, 18, 19, 20, 21, 22, 23, 24, 25, 26, 27, 28, 29, 30, 31, 32, 33, 34, 35, 36, 37, 38, 39, 40, 41, 42, 43, 44, 45, 46, 47, 48, 49, 50, 51, 52, 53, 54, 55, 56, 57, 58, 59, 60, 61, 62, 63, 64, 65, 66, 67, 68, 69, 70, 71, 72, 73, 74, 75, 76, 77, 78, 79]). It should be noted that Table 1 was drafted in an effort to balance the trade-off between accuracy of findings and simplicity for the average reader. Some studies have conducted several different analyses (severe vs critical disease, mild vs severe disease, mild vs critical disease, severe vs non-severe disease, survivors vs non-survivors etc.) including a different number of included primary studies in each analysis and subsequently different number of included patients. All these data could not possibly be presented in detail as the aim of Table 1 is to provide a rough overview of the literature while trying not to be exhaustive or reader unfriendly. The main and most important findings of our systematic review are plotted in the graphical summary presented in Fig. 2.

**Table 1.** **Meta-analyses on cardiac injury in hospitalized patients with COVID-19**.

Study	Study outcome	Literature search (until dd.mm.yyyy)	N of included studies	N of patients	Cardiac injury definition	Main Findings (95% CI)
Ogungbe *et al*. [52]	∙ Incidence of CI	08.2021		21367 (incidence)	Troponin I or T	∙ Incidence 18.5% (17.9, 19.0)
	∙ CI and severity		16 (OR)			∙ Severity OR 1.93 (1.45, 2.40)/HR 1.75 (1.48, 2.10)
	∙ CI and mortality		20 (HR)			∙ Mortality OR 1.72 (1.32, 2.25)/HR 1.51 (1.31, 1.75)
Changal *et al*. [51]	∙ Incidence of CI	01.05.2020	7	12577	Troponin I or T	∙ Incidence 27.2% (9.2–51)
	∙ CI and mortality					∙ Mortality HR 2.43 (2.28, 3.60)
Ma *et al*. [50]	∙ Incidence of CI	08.09.2021	60 (incidence)	50284 (incidence)	Troponin I	∙ Incidence 22.9% (19, 27)
	∙ CI and severity					∙ Severity OR 7.06 (3.94, 12.65)
	∙ CI and mortality					∙ Mortality OR 7.75 (4.4, 13.66)
An *et al*. [49]	∙ CI and severity	09.05.2021	28 (severity)	7812 (severity)	Troponin	∙ Severity SMD 0.81 U/L (0.14, 1.48)
	∙ CI and mortality		41 (mortality)	9532 (mortality)		∙ Mortality SMD 0.51 U/L (0.37, 0.64)
Zhu *et al*. [48]	∙ CI and mortality	27.06.2020	9	NR	Troponin I	∙ Mortality RR 7.01 (5.64, 8.71)
	∙ CI and severity					∙ Severity OR 1.76 (0.61, 5.07)
						∙ Severity SMD 0.48 (–0.18, 1.14)
Abate *et al*. [47]	∙ Incidence of CI	05.2021	37 (incidence)	21204 (incidence)	Depending on each study	∙ Incidence 22.3% (17.9, 26.8)
	∙ CI and mortality		15 (mortality)			∙ Mortality OR 8.12 (5.19, 12.71)
Katzenschlager *et al*. [46]	∙ CI and mortality	31.05.2020	13	NR	Troponin I	∙ DoM 21.88 pg/mL (9.78, 33.99)
Zinellu *et al*. [45]	∙ CI and severity	01.2021	55	11791	CK-MB	∙ SMD 0.81 (0.61, 1.01)
	∙ CI and mortality					∙ RR 2.84 (1.89, 4.27)
Long *et al*. [44]	∙ CI and mortality	09.11.2020	12	2708	High-sensitive Troponin I or T	∙ OR 13.25 (8.56, 20.52)
Wu *et al*. [43]	∙ CI and mortality	26.05.2020	4 (MD)	NR	Troponin	∙ MD 66.65 pg/mL (16.94, 116.36)
			6 (OR)			∙ OR 25.16 (6.56, 96.44)
Patel *et al*. [42]	∙ Incidence of CI	30.06.2020	11	1361	Depending on each study	∙ Incidence 14.5% (12.7, 16.4)
	∙ CI and mortality					∙ OR 9.93 (3.95–25.0)
Cheng *et al*. [41]	∙ Incidence of CI	29.08.2020	57	34072	Troponin	∙ Incidence 21% (18, 26)
Toloui *et al*. [40]	∙ Incidence of CI	30.04.2020	26 (incidence)	NR	Depending on each study	∙ Incidence 19.5% (18.23, 20.72)
	∙ CI and mortality		14 (mortality)			∙ OR 14.24 (8.67–23.38)
Alzahrani *et al*. [39]	∙ Incidence of CI	11.04.2020	7	1380	Troponin I or T	∙ Incidence 20.6% (17.2, 24.0)
	∙ CI and mortality					∙ SMD 2.15 (0.83, 3.47)
						∙ RR 5.28 (3.71, 7.51)
Zhao *et al*. [38]	∙ Incidence of CI	30.11.2020	8	NR	Depending on each study	∙ Incidence 21.2% (12.3, 30.0)
Tiruneh *et al*. [37]	∙ Incidence of CI	10.04.2020	14	1215	Depending on each study	∙ Incidence 6.4% (2.8, 15.6)
Kansestani *et al*. [53]	∙ CI and severity	30.07.2020	7 (severity)	1142 (severity)	Troponin I	∙ Sensitivity/specificity for critical/noncritical and survivors/non-survivors prognosis 0.35/0.94 and 0.59/0.88, respectively
	∙ CI and mortality		8 (mortality)	4054 (mortality)		
Dalia *et al*. [36]	∙ CI and severity	07.06.2020	14	3623	Troponin I	∙ MD 77.9 pg/mL (–6.47, 162.33)
Wungu *et al*. [35]	∙ CI and severity	08.2020	7 (severity)	1163 (severity)	Troponin	∙ Severity SMD 0.77 (–0.37, 1.92)
	∙ CI and mortality		9 (mortality)	3886 (mortality)		∙ Mortality SMD 1.64 (0.83, 2.45)
Qiang *et al*. [34]	∙ CI and severity	NA	NA	NA	NA	∙ OR 11.83
Wibowo *et al*. [33]	∙ Incidence of CI	16.12.2020	13	12262	Troponin	∙ Incidence 31% (23–38)
	∙ CI and mortality					∙ OR 4.75 (4.07, 5.53)
Sheth *et al*. [32]	∙ CI and severity	15.04.2020	15	1715	Troponin	∙ Severity WMD 0.28 (–0.14, 0.69)
	∙ CI and mortality					∙ Mortality WMD 0.61 (0.46–0.76)
						∙ Mortality OR 6.641 (1.26, 35.1)
Sahranavard *et al*. [31]	∙ Incidence of CI	16.04.2020	13 (incidence)	NR	Troponin I	∙ Incidence 17.85% (13.18–23.72)
	∙ CI and mortality		4 (mortality)			∙ MD 31.8 pg/mL (17.9, 45.7)
Koeppen *et al*. [30]	∙ Incidence of CI	25.11.2020	14	927	Depending on each study	∙ Incidence 30% (19, 42)
Chaudhary *et al*. [29]	∙ CI and severity	11.07.2020	18	3375	Depending on each study	∙ WMD 10.69 (7.02, 14.36)
	∙ CI and mortality					
Zhong *et al*. [28]	∙ Incidence of CI	12.04.2020	15	1118	Depending on each study	∙ Incidence 37.1% (27.4-47.4)
Mudatsir *et al*. [27]	∙ CI and severity	04.04.2020	6	530	High-sensitive Troponin I	∙ OR 9.25 (3.51, 24.37)
						∙ SMD 1.22 (0.69, 1.74)
Zhang *et al*. [26]	∙ CI and severity	10.04.2020	4	612	Depending on each study	∙ OR 6.35 (1.22, 33.14)
Hu *et al*. [25]	∙ Incidence of CI	26.07.2020	7 (incidence)	NR	Troponin I or T	∙ Incidence 29% (13, 45)
	∙ CI and severity					∙ OR 4.71 (2.23, 9.92)
Fu *et al*. [24]	∙ Incidence of CI	07.2020	21 (incidence)	6297 (incidence)	Depending on each study	∙ Incidence 22% (16, 28)
	∙ CI and mortality		10 (mortality)			∙ OR 10.11 (4.49, 22.77)
Zhao *et al*. [23]	∙ Incidence of CI	15.10.2020	35 (incidence)	22473 (incidence)	Troponin	∙ Incidence 20.8% (16.8, 25.0)
	∙ CI and mortality		11 (mortality)	13889 (mortality)		∙ Adjusted RR 2.68 (2.08, 3.46)
Hessami *et al*. [22]	∙ Incidence of CI	27.05.2020	6 (incidence)	NR	Depending on each study	∙ Incidence 15.6% (5.15, 47.12)
	∙ CI and mortality		12 (mortality)			∙ OR 13.29 (7.35, 24.03)
Malik *et al*. [21]	∙ CI and severity	15.08.2020	10	3982	Hypersensitive Troponin I	∙ OR 7.92 (3.70, 16.97)
Huang *et al*. [20]	∙ Incidence of CI	05.06.2020	43 (incidence)	9475 (incidence)	Depending on each study	∙ Incidence 19% (15, 22)
	∙ CI and mortality		20 (mortality)			∙ Pooled ES 4.99 (3.38, 7.37)
Vakhshoori *et al*. [19]	∙ Incidence of CI	25.03.2020	7	970	Troponin, electrocardiography, echocardiography	∙ Incidence 15% (11, 20)
Bansal *et al*. [18]	∙ CI and mortality	17.06.2020	8	1609	Depending on each study	∙ RR 7.79 (4.69, 13.01)
Mesas *et al*. [17]	∙ CI and mortality	27.07.2020	15	NR	Troponin	∙ MD 0.02 ng/mL (0.02, 0.02)
						∙ Pooled ES 0.91 (0.13, 1.70)
Prasitlumkum *et al*. [16]	∙ Incidence of CI	08.2020	27	8971	Depending on each study	∙ Incidence 20% (16.1, 23.8)
Zeng *et al*. [15]	∙ CI and mortality	02.05.2020	NR	NR	Troponin	∙ RR 4.89 (3.84, 6.22)
Walker *et al*. [14]	∙ CI and severity	10.07.2020	22	4468	Troponin I, CK-MB	∙ MD 0.54 ng/mL (0.36, 0.72) (troponin)
Vakili *et al*. [13]	∙ Incidence of CI	01.05.2020	15	NR	Depending on each study	∙ Incidence 15.68% (11.1, 20.97)
Luo *et al*. [12]	∙ CI and severity	07.2020	11 (incidence)	NR	Depending on each study	∙ Severity OR 6.57 (3.7, 11.65)
	∙ CI and mortality		14 (mortality)			∙ Mortality OR 11.03 (6.74, 18.05)
Moutchia *et al*. [11]	∙ CI and severity	18.04.2022	8	2379	Troponin I	∙ MMD 0.01 ng/ml (0.00, 0.02)
Gu *et al*. [1]	∙ Incidence of CI	24.04..2020	53	7679	Troponin, CK-MB, electrocardiography, echocardiography	∙ Incidence 21% (17, 25)
Shoar *et al*. [5]	∙ CI and mortality	15.03.2020	12	1845	Depending on each study	∙ OR 20.3 (7.8, 53.3)
Danwang *et al*. [79]	∙ CI and severity	18.04.2020	4 (CK-MB)	1150 (CK-MB)	Troponin I, CK-MB	∙ SMD 0.68 (0.48, 0.87) (CK-MB)
			2 (Troponin-I)	430 (Troponin I)		∙ SMD 0.71 (0.42, 1.00) (Troponin I)
Zou *et al*. [78]	∙ Incidence of CI	30.05.2020	16	2224	Troponin	∙ Incidence 24.4% (22.6, 26.2)
	∙ CI and mortality					∙ OR 17.83 (10.89, 29.21)
Sanz-Sánchez *et al*. [77]	∙ CI and mortality	08.07.2020	14	6462	Depending on each study	∙ OR 9.16 (5.30, 15.83)
						∙ Adjusted HR 1.62 (1.35, 1.94) (data from 4 studies)
Khinda *et al*. [76]	∙ CI and severity	01.05.2020	50 (severity)	11173 (severity)	High-sensitive Troponin I	∙ Severity WMD 11.07 pg/mL (3.64, 18.50)
	∙ CI and mortality		15 (mortality)	2525 (mortality)		∙ Mortality WMD 90.47 pg/mL (47.79, 133.14)
Wu *et al*. [10]	∙ CI and severity	13.05.2020	NR	NR	High-sensitive Troponin I	∙ WMD 15.99 pg/mL (6.24, 25.74)
Ghahramani *et al*. [75]	∙ CI and severity	03.03.2020	5	3396	Troponin I	∙ SMD 0.27 (–0.14, ${\mathrm{0.67)}}^{*}$
Zuin *et al*. [74]	∙ Incidence of CI	10.04.2020	9	1686	Depending on each study	∙ Incidence 23.9% (21.9, 26.1)
	∙ CI and mortality					∙ OR 21.6 (8.6, 54.4)
Li *et al*. [73]	∙ CI and severity	30.03.2020	9	1548	Troponin I	∙ Severity RR 5.57 (3.04, 10.22)
	∙ CI and mortality					∙ Mortality RR 5.64 (2.69, 11.83)
Zhao *et al*. [72]	∙ CI and severity	08.02.2020	2	179	Depending on each study	∙ RR 10.32 (3.05, 34.96)
De Lorenzo *et al*. [71]	∙ Incidence of CI	04.02.2020	8	1229	Troponin	∙ Incidence 16% (9, 27)
Momtazmanesh *et al*. [70]	∙ Incidence of CI	21.04.2020	16	2647	Depending on each study	∙ Incidence 25.3% (19.5, 31.1)
	∙ CI and severity					∙ Severity OR 6.28 (4.22, 9.80) (17 studies)
	∙ CI and mortality					∙ Mortality OR 19.64 (10.28, 37.53)
Liu *et al*. [69]	∙ Incidence of CI	22.05.2020	26	4753	Depending on each study	∙ Incidence 13% (8, 18)
Potere *et al*. [68]	∙ Incidence of CI	10.04.2020	10 (peer- reviewed)	2389	Depending on each study	∙ Incidence 15% (11, 20), peer reviewed studies
			10 (not peer-reviewed)			∙ Incidence 5% (2, 10), not peer reviewed studies
Parohan *et al*. [67]	∙ CI and severity	20.05.2020	4 (severity)	852 (severity)	Troponin I	∙ Severity WMD 4.05 pg/mL (–0.20, ${\mathrm{8.30)}}^{*}$
	∙ CI and mortality		3 (mortality)	1230 (mortality)		∙ Mortality WMD 26.35 pg/mL (14.54, 38.15)
Huang *et al*. [4]	∙ CI and severity	12.02.2020	2	179	Depending on each study	∙ OR 13.48 (3.60, 50.47) for CI in severe vs non-severe
Vrsalovic *et al*. [66]	∙ CI and mortality	NR	2	940	High-sensitive Troponin I	∙ Adjusted HR 3.08 (1.95, 4.87)
Kollias *et al*. [65]	∙ CI and mortality	30.05.2020	3	3956	High-sensitive Troponin I	∙ Adjusted HR 2.3 (1.3, 4.1)
Toraih *et al*. [64]	∙ CI and severity/mortality	08.05.2020	31	32	Troponin I	∙ OR 5.22 (3.73, ${\mathrm{7.31)}}^{†}$
Aikawa *et al*. [63]	∙ CI and mortality	13.04.2020	6	1231	High-sensitive Troponin	∙ OR 22.7 (13.6, 38.1)
Li *et al*. [62]	∙ CI and mortality	14.04.2020	8	1429	Depending on each study	∙ OR 21.15 (10.19, 43.94)
Tian *et al*. [61]	∙ CI and mortality	24.04.2020	3	615	High-sensitive Troponin I	∙ WMD 44.2 ng/L (19.0, 69.4)
Vrsalovic *et al*. [60]	∙ CI and mortality	30.04.2020	3	803	High-sensitive Troponin I	∙ Adjusted HR 2.48 (1.50, 4.11)
Shao *et al*. [59]	∙ CI and mortality	30.03.2020	9	1470	Troponin	∙ OR 13.68 (9.81, 19.08)
Zheng *et al*. [58]	∙ CI and severity/mortality	20.03.2020	2	186	High-sensitive Troponin I	∙ OR 43.24 (9.92, 188.49) for CI in severe/death vs non-severe
Santoso *et al*. [57]	∙ CI and severity	29.03.2020	3 (severity)	622 (severity)	High-sensitive Troponin I	∙ Severity RR 13.81 (5.52, 34.52)
	∙ CI and mortality		7 (mortality)	1550 (mortality)		∙ Mortality RR 7.95 (5.12, 12.34)
Li *et al*. [56]	∙ CI and severity	27.03.2020	14 (severity)	NR	Troponin, electrocardiography, echocardiography	∙ Severity SMD 0.53 (0.30, 0.75) (troponin)
	∙ CI and mortality		9 (mortality)			∙ Mortality RR 3.85 (2.13, 6.96)
Lippi *et al*. [55]	∙ CI and severity	04.03.2020	4	341	Troponin I	∙ SMD 25.6 ng/L (6.8, 44.5)
Li *et al*. [54]	∙ Incidence of CI	02.2020	2	179	Depending on each study	∙ Incidence 8% (4, 12)

CI, cardiac injury; CIs, confidence intervals; CK-MB, Creatine Kinase-MB; DoM, difference of medians; ES, effect size; HR, hazard ratio; hsTropI, high sensitive Troponin I; ICU, intensive care unit; MD, mean difference; MMD, meta-mean difference; NA, data not available (no full text available); NR, not reported; OR, odds ratio; RR, relative risk; SMD, Standardized mean difference; WMD, weighted mean difference. ^*^not statistically significant. ^†^conversion of SMD to OR.

Not any meta-analysis was excluded due to non-English language. The majority of studies (46 studies) reported the impact of CI on mortality (Table 1) [5, 12, 15, 17, 18, 20, 22, 23, 24, 29, 31, 32, 33, 35, 39, 40, 42, 43, 44, 45, 46, 47, 48, 49, 50, 51, 52, 53, 56, 57, 58, 59, 60, 61, 62, 63, 64, 65, 66, 67, 70, 73, 74, 76, 77, 78]. Among the different statistical indices, OR was the most frequently used (29 studies) [4, 5, 12, 21, 22, 24, 25, 26, 27, 32, 33, 34, 40, 42, 43, 44, 47, 48, 50, 52, 58, 59, 62, 63, 64, 70, 74, 77, 78], while RR and HR were used in 10 [15, 18, 23, 39, 45, 48, 56, 57, 72, 73] and 6 studies [51, 52, 60, 65, 66, 77], respectively. A minority of meta-analyses investigated pooled differences of cardiac biomarkers between severe vs non-severe disease or survivors vs non survivors (Table 1). Certain meta-analyses included primary research papers that had not undergone peer review process [68], while two meta-analyses were not initially peer-reviewed [80, 81]. However, both these meta-analyses were later formally peer-reviewed and published, and thus they were finally included in our systematic review [32, 46]. In most meta-analyses, Chinese studies were the main source of evidence [16, 45, 64, 79], some of them available only in Chinese language. Zinellu *et al*. [45] performed a meta-analysis of 55 studies (including 11,791 hospitalized patients with COVID-19), aiming to investigate the association of CI (defined as elevated creatine kinase-MB levels) with COVID-19 severity and subsequent mortality. Among the included studies in this meta-analysis, 95% (n = 52) were conducted in China [45]. Similarly in a meta-analysis of 27 studies investigating the incidence of CI among patients with COVID-19, 81% of the included studies (n = 22) were conducted in China [16].

### 3.1 Definition of CI

Among the included meta-analyses, several different definitions were used as inclusion criteria for studies reporting CI in patients with COVID-19. Some meta-analyses required CI definition to be based on high-sensitive troponin I [76], while others did not have strict limitations and included studies with different CI definitions [5]. It should be noted that definitions were mainly based on cardiac biomarkers kinetics, while rarely included electrocardiographic and/or echocardiographic findings additionally assessed and used for CI definition (Table 1).

### 3.2. Sample Size—Literature Search Date

Sample size varied across the included meta-analyses. Ma *et al*. [50] reported CI incidence of about 23% among 50284 hospitalized patients with COVID-19. On the other hand, Li *et al*. [54] (one of the earliest meta-analyses including 2 studies) reported the incidence of CI (8%) among a sample of 179 hospitalized patients with COVID-19. Interestingly, the number of patients was significantly greater in studies (or different sub-analyses within the same study) evaluating the incidence and epidemiology of CI than in studies/analyses investigating the prognostic value of CI [31, 40, 41, 47, 50] (Table 1).

Literature search dates varied from study to study and were directly associated with the time of publication. The most updated literature search was included in the meta-analysis by Ma *et al*. [50] and corresponds to literature search until September 2021.

### 3.3 Incidence of CI among Hospitalized Patients with COVID-19

30 meta-analyses reported the incidence of CI among hospitalized patients with COVID-19 [1, 13, 16, 19, 20, 22, 23, 24, 25, 28, 30, 31, 33, 37, 38, 39, 40, 41, 42, 47, 50, 51, 52, 54, 68, 69, 70, 71, 74, 78]. The estimated pooled incidence ranged across meta-analyses from 5% [68] to 37% [28]. However, the 5% finding was not based on peer-reviewed evidence and might be subject to limitations [68].

### 3.4 CI Impact on COVID-19 Severity

32 studies reported on the impact of CI on COVID-19 infection severity or the difference in cardiac biomarkers levels between patients with severe vs non-severe COVID-19 infection [4, 10, 11, 12, 14, 21, 25, 26, 27, 29, 32, 34, 35, 36, 45, 48, 49, 50, 52, 53, 55, 56, 57, 58, 64, 67, 70, 72, 73, 75, 76, 79]. Statistical indices used were OR and RR for impact on outcome evaluation and SMD and WMD for difference in biomarkers levels evaluation (Table 1). Nearly all studies demonstrated significant associations between CI and severity of COVID-19 infection. Only 6 studies failed to show a significant association [32, 35, 36, 48, 67, 75].

### 3.5 CI impact on COVID-19 Mortality

46 meta-analyses analyzed the impact of CI on COVID-19 mortality. Statistical indices most commonly used were OR, HR and RR. Interestingly adjusted HR was used only in 4 meta-analyses [60, 65, 66, 77]. WMD was also used, to provide difference in biomarkers levels among COVID-19 survivors vs non-survivors. All studies demonstrated a significant effect of CI on COVID-19 infection mortality. OR ranged from 1.72 to 43.24, however it was found to be about 10–15 in most of the included meta-analyses (Table 1). Adjusted HR used in four meta-analyses as reported above [60, 65, 66, 77], which is generally more representative of the reality due to adjustment for several confounding factors, barely passed the value 3.

## 4. Discussion

The aim of the present systematic review was to identify all meta-analyses that have been conducted regarding the incidence and impact of CI in hospitalized patients with COVID-19. An impressive number of 73 meta-analyses were identified. 46 and 32 meta-analyses investigated the impact of CI on COVID-19 mortality and disease severity, respectively. 30 meta-analyses investigated CI incidence among hospitalized patients with COVID-19. The majority of meta-analyses demonstrated the prognostic value of CI and the association with poor COVID-19 related outcomes. The incidence of CI presented significant heterogeneity among included analyses (from 5 to 37%), however it should be emphasized that CI was demonstrated not to be a rare clinical condition.

After a systematic literature search, four potentially relevant meta-analyses were excluded: three on pediatric populations [82, 83, 84] and one dealing exclusively with the impact of N-terminal pro-brain natriuretic peptide level on COVID-19 mortality [85]. Among the finally included meta-analyses, those on CI and COVID-19 severity/mortality were arithmetically more numerous, yet with significantly smaller sample sizes compared with those evaluating the incidence of CI. Patients included in the analyses were hospitalized patients with COVID-19 with different levels of disease severity (e.g., meta-analyses of studies in general wards or ICU). The heterogeneity of reported incidence could be partially attributed to this fact. Additionally, literature search dates varied from meta-analysis to meta-analysis and were directly linked to the publication date of each paper. The time period difference between literature and publication dates mainly depicts timing issues of publication procedures of the involved journals. Regarding the robustness of the conclusions of the included meta-analysis, it should be noted that the vast majority were in line regarding a harmful effect of CI on COVID-19 severity and mortality. Only a few studies evaluating COVID-19 severity failed to demonstrate this association. Severity assessment may present differences and discrepancies among different analyses, however the harmful effect of CI was concretely demonstrated when death—the ultimate hard endpoint—was considered.

Among meta-analyses and primary research studies included in these meta-analyses there is significant heterogeneity concerning a variety of methodological aspects [65]: (i) some meta-analyses often include studies from the same hospital. In this way data derived from overlapping populations are introduced into the analysis. This is always a tricky and crucial part when conducting a meta-analysis. In case of same hospital studies, communication with all corresponding authors should ensure that included studies do not include overlapping populations, leading to over- or underestimation of findings; (ii) certain meta-analyses include only primary research studies from China [16, 45, 67]. Generalisation of conclusions should be carefully and critically considered; (iii) inclusion of studies that have not been subjected to peer-review process. COVID-19 pandemic has led to many papers’ fast track publication. This has been in part inevitable due to the urgent need for data. However, through years of research we have gained experience and understood that even peer-review process may not be enough to guarantee the quality of a published study, yet it is the best tool we currently possess. Thus, while not peer reviewed studies are obviously useful, they should be interpreted with caution. Interestingly, Potere *et al*. [68] performed two different analyses including peer-reviewed and not-peer reviewed articles in order to assess CI incidence among hospitalised patients with COVID-19. In the first case he reported an incidence of 15%, while in the second case an incidence of 5% (Table 1); (iv) all 73 included articles were written in English language. However, primary research papers written in Chinese, were included in some meta-analyses, rendering the reproduction of their results difficult [56]. This was perhaps unavoidable given that the vast majority of data, at least primarily, came from China, specifically Wuhan hospitals; (v) the definition of CI varied across included studies. Some meta-analyses included studies that based their definition strictly on high-sensitivity troponin I, while others included studies with different definitions of CI (e.g., based on non-high-sensitivity troponin, more than one cardiac biomarker, echocardiography etc.) (Table 1); (vi) CI is based on observations and measurements mainly upon admission, but this is not totally clear in some studies; (vii) Depending on the study, CI was expressed as a continuous (troponin level) or dichotomous (based on troponin cutoffs) variable; (viii) different statistical indices have been used to describe CI impact on COVID-19 prognosis. OR used across studies has been mainly an unadjusted index, while only a few studies used adjusted HR for possible confounders to quantify CI and COVID-19 association [60, 65, 66, 77].

CI is a multifactorial phenomenon in COVID-19 infection. As reported above many different mechanisms are implicated, most frequently not related to atherosclerosis [7]. This observation led the American College of Cardiology to recommend troponin not to be measured as a routine in all patients with COVID-19, as in most cases high values would falsely lead to acute coronary syndrome work-up. Interestingly, among 73 meta-analyses identified on this topic [1, 4, 5, 10, 11, 12, 13, 14, 15, 16, 17, 18, 19, 20, 21, 22, 23, 24, 25, 26, 27, 28, 29, 30, 31, 32, 33, 34, 35, 36, 37, 38, 39, 40, 41, 42, 43, 44, 45, 46, 47, 48, 49, 50, 51, 52, 53, 54, 55, 56, 57, 58, 59, 60, 61, 62, 63, 64, 65, 66, 67, 68, 69, 70, 71, 72, 73, 74, 75, 76, 77, 78, 79, 81], the terms ST Elevation Myocardial Infarction (STEMI) or non-ST Elevation Myocardial Infarction (NSTEMI) were never encountered. What seems more reasonable is the view of Chapman *et al*. [7]. Although they recognize the potentially problematic use of troponin in patients with COVID-19, they cannot disregard its important prognostic value [7]. Physicians should be taught to better interpret laboratory tests, rather than abstain from ordering them [7]. Based on the above and according to our experience in our Reference Center, an initial assessment of troponin level and CI at least once upon admission may be reasonable in most patients with COVID-19. Future studies may shed light on different diagnostic approaches in patients with COVID-19. Moreover, troponin thresholds for STEMI or NSTEMI diagnosis may not be the same in patients with or without COVID-19.

**Fig. 3.**
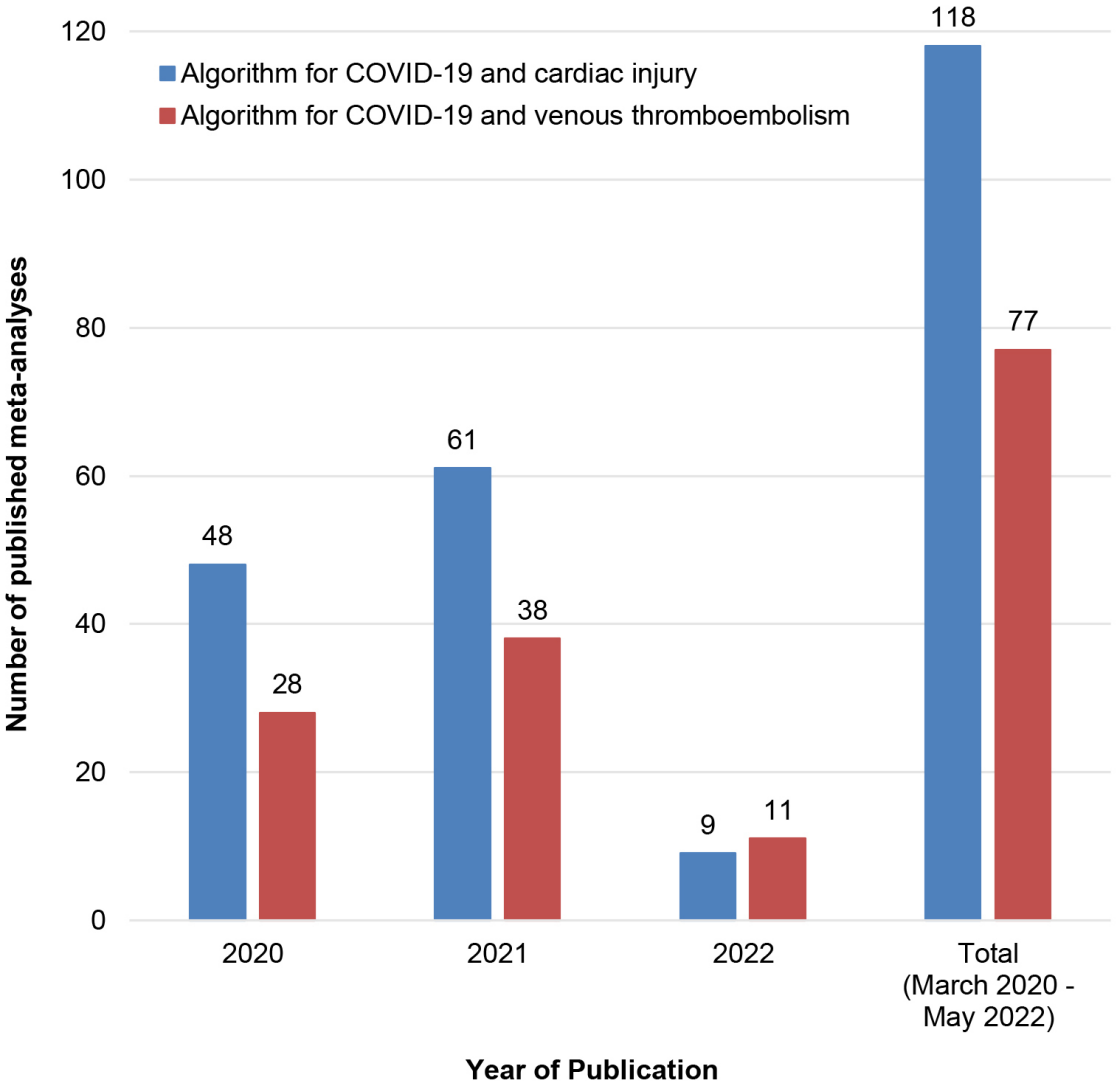
**Comparison of results retrieved after PubMed search for COVID-19 and cardiac injury or COVID-19 and venous thromboembolism**. Search algorithms: *COVID-19 and cardiac injury*: (“coronavirus 2019” OR “2019-nCoV” OR “SARS-CoV-2” OR “COVID-19” OR “coronavirus disease 2019”) AND (troponin OR “cardiac injury” OR “myocardial injury”) AND (“meta-analysis” OR meta analysis); *COVID-19 and venous thromboembolism*: (“coronavirus 2019” OR “2019-nCoV” OR “SARS-CoV-2” OR “COVID-19” OR “coronavirus disease 2019”) AND (“deep vein” OR “pulmonary embolism” OR “venous thromboembolism”) AND (“meta-analysis” OR meta analysis).

The first meta-analysis on CI and COVID-19 was published in March 2020 by Li *et al*. [54], about 3 months after the onset of the pandemic in China. The present systematic review conducted a systematic literature search until May 2022 and identified 73 meta-analyses regarding the association of CI and COVID-19. This could be translated into 73 meta-analyses within 26 months or nearly 1 meta-analysis every ten days! Surprisingly, after performing systematic literature search to identify meta-analyses on the association of venous thromboembolism and COVID-19 (maybe the most popular COVID-19 related topic—at least before the distribution of vaccines), only 77 articles were retrieved (Fig. 3). After considering this comparison, the interest and the potential of authors in performing meta-analyses on CI and COVID-19 becomes even more impressive.

## 5. Conclusions

Overcoming the important methodological inconsistencies, the main conclusion of all meta-analyses is that CI is not rare and is indisputably associated with worse outcomes in hospitalized patients with COVID-19. Multiple pathophysiological mechanisms are implicated, and more careful diagnostic approach of elevated troponin level should be enhanced. The aforementioned chaotic heterogeneity and diversity of studies could be interpreted as making these observations even more robust, as different studies, different methodologies and different authors reach similar conclusions. CI incidence and impact on COVID-19 prognosis may have been one of the most meta-analysed topics of our days.
